# Prevention of Acute Kidney Injury by Tauroursodeoxycholic Acid in Rat and Cell Culture Models

**DOI:** 10.1371/journal.pone.0048950

**Published:** 2012-11-09

**Authors:** Sandeep Gupta, Shunan Li, Md. Joynal Abedin, Kajohnsak Noppakun, Lawrence Wang, Tarundeep Kaur, Behzad Najafian, Cecília M. P. Rodrigues, Clifford J. Steer

**Affiliations:** 1 Department of Medicine, University of Minnesota Medical School, Minneapolis, Minnesota, United States of America; 2 Department of Laboratory Medicine and Pathology, University of Minnesota Medical School, Minneapolis, Minnesota, United States of America; 3 Research Institute for Medicines and Pharmaceutical Sciences (iMed.UL), Faculty of Pharmacy, University of Lisbon, Lisbon, Portugal; 4 Department of Genetics, Cell Biology and Development, University of Minnesota Medical School, Minneapolis, Minnesota, United States of America; Massachusetts Eye & Ear Infirmary, Harvard Medical School, United States of America

## Abstract

**Background:**

Acute kidney injury (AKI) has grave short- and long-term consequences. Often the onset of AKI is predictable, such as following surgery that compromises blood flow to the kidney. Even in such situations, present therapies cannot prevent AKI. As apoptosis is a major form of cell death following AKI, we determined the efficacy and mechanisms of action of tauroursodeoxycholic acid (TUDCA), a molecule with potent anti-apoptotic and pro-survival properties, in prevention of AKI in rat and cell culture models. TUDCA is particularly attractive from a translational standpoint, as it has a proven safety record in animals and humans.

**Methodology/Principal Findings:**

We chose an ischemia-reperfusion model in rats to simulate AKI in native kidneys, and a human kidney cell culture model to simulate AKI associated with cryopreservation in transplanted kidneys. TUDCA significantly ameliorated AKI in the test models due to inhibition of the mitochondrial pathway of apoptosis and upregulation of survival pathways.

**Conclusions:**

This study sets the stage for testing TUDCA in future clinical trials for prevention of AKI, an area that needs urgent attention due to lack of effective therapies.

## Introduction

Kidneys are acutely injured when deprived of nutrients and exposed to nephrotoxins. Acute kidney injury (AKI) has reached epidemic proportions and has grave short- and long-term consequences on patient health and cost of care [1]. Even kidneys that regain normal function following AKI have persistent maladaptive alterations that may result in a higher incidence of hypertension and chronic kidney disease [2]–[5]. Even in situations where the onset of AKI is predictable, such as perioperative kidney injury, none of the current therapies can prevent AKI. Thus, there is a critical need to develop therapies for the prevention of AKI.

Following acute kidney injury, cells die either immediately by necrosis or over hours to days by apoptosis, or programmed cell death. Cells under stress resist death by upregulating survival pathways. AKI can be prevented under experimental conditions by upregulating survival pathways by pro-survival molecules such as Survivin [6] or by ischemic preconditioning [7], [8]. Similarly, anti-apoptotic molecules have been shown to prevent AKI in animal models [9], [10]. However, these experimental approaches are limited in their translational potential by toxicity. Therefore, an ideal therapy for prevention of AKI should be nontoxic, pro-survival, and anti-apoptotic.

The liver may provide clues for developing such a therapy for AKI. Liver cells are exposed to toxic compounds and have well-developed cytoprotective mechanisms. Of the known mechanisms, protection by ursodeoxycholic acid (UDCA) and its taurine conjugate, tauroursodeoxycholic acid (TUDCA), has been well studied. U/TUDCA prevent cell death by stabilizing the cell membranes, inhibiting apoptosis, and upregulating survival pathways [11]–[15]. Furthermore, protection by U/TUDCA extends beyond liver to other cells in the body [16]–[18]. For example, hibernating animals such as black bears have high blood levels of UDCA, which prevents cell death under low nutrient conditions encountered during long periods of hibernation [19], [20]. In contrast, humans have very low blood levels of UDCA. Black bear bile has been used in traditional Chinese medicine for more than 3000 years; and western medicine is increasingly recognizing the therapeutic value of U/TUDCA. U/TUDCA have been used effectively for treating human liver diseases [21]–[26], usually at dosages up to 20 mg/kg/day orally for long periods. In experimental models of acute injury such as myocardial infarction [16], stroke [17], [18], and spinal cord injury [27]–[29], dosages as high as 500 mg/kg/day were administered intraperitonially or intravenously as single or short period injections. Furthermore, several studies have shown U/TUDCA to be safe for animal [16], [18], [29], [30] and human applications [22], [31], making them attractive molecules from a translational standpoint.

AKI is often predictable in clinical situations such as following surgery; exposure to nephrotoxic medications; and donor nephrectomy during cryopreservation. However, none of the current therapies can prevent AKI. Our vision in planning these studies was to develop a therapy with high translational potential that can be administered for prevention of AKI. Thus in this study we tested our hypothesis that TUDCA can prevent AKI. We chose TUDCA over UDCA because of its higher solubility at physiological pH, a characteristic that permits rapid parenteral administration in high doses and avoids precipitation during cryopreservation of donor kidneys. Accordingly, in this study we determined the efficacy and mechanisms of action of TUDCA in a rat model of AKI and a human kidney cell culture model of cryopreservation injury.

## Results

### *In vivo* Experiments

#### Functional protection

**Figure 1:**
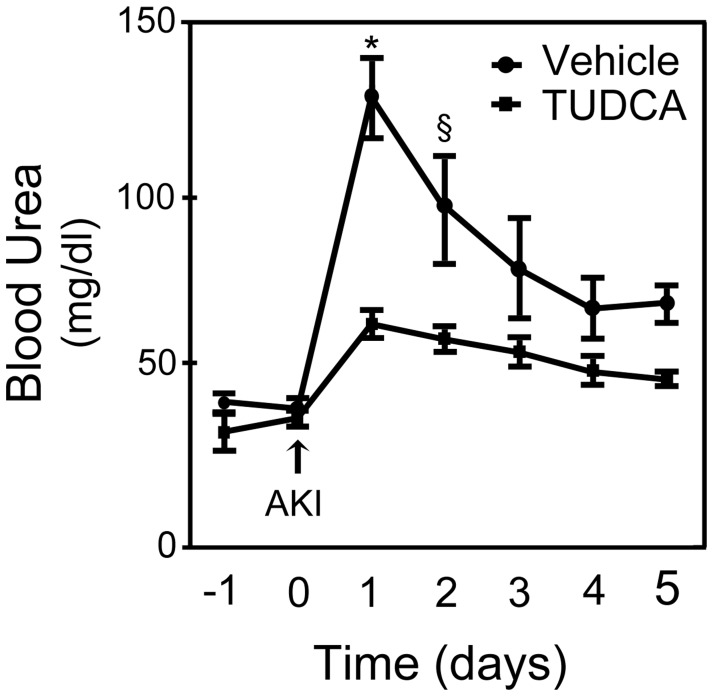
Functional protection against AKI by TUDCA. Daily administration of 400 mg/kg of TUDCA protected against ischemic AKI. Renal function was determined by daily measurement of blood urea. Rats that received TUDCA (squares) had significantly less elevation in blood urea levels on day 1 and day 2 following ischemia-reperfusion injury as compared to rats that received vehicle (circles) (*p*<0.01). Similarly, on days 3–5, blood urea levels continued to remain lower in the TUDCA-treated rats; however, the difference was not statistically significant. Results are expressed as mean ± standard deviation of a least 3 different animals in each group. *p<0.001 and §*p*<0.01 from day 0.

Rats were given 400 mg/kg/day of TUDCA or equal volume of vehicle from three days before until five days following the induction of AKI. Renal function was determined by daily measurements of blood urea levels. Rats in the TUDCA group had significantly less elevation in blood urea levels on days 1 (*p*<0.001) and 2 (*p*<0.01) following AKI as compared to those in the vehicle group (Figure 1). Although on days 3–5 blood urea was lower in the TUDCA-treated rats, the difference was not statistically significant. Interestingly, the blood urea continued to decline in the TUDCA-treated group until the day of euthanasia (day 5), while it stabilized above baseline in the vehicle-treated group.

#### Structural protection

**Figure 2:**
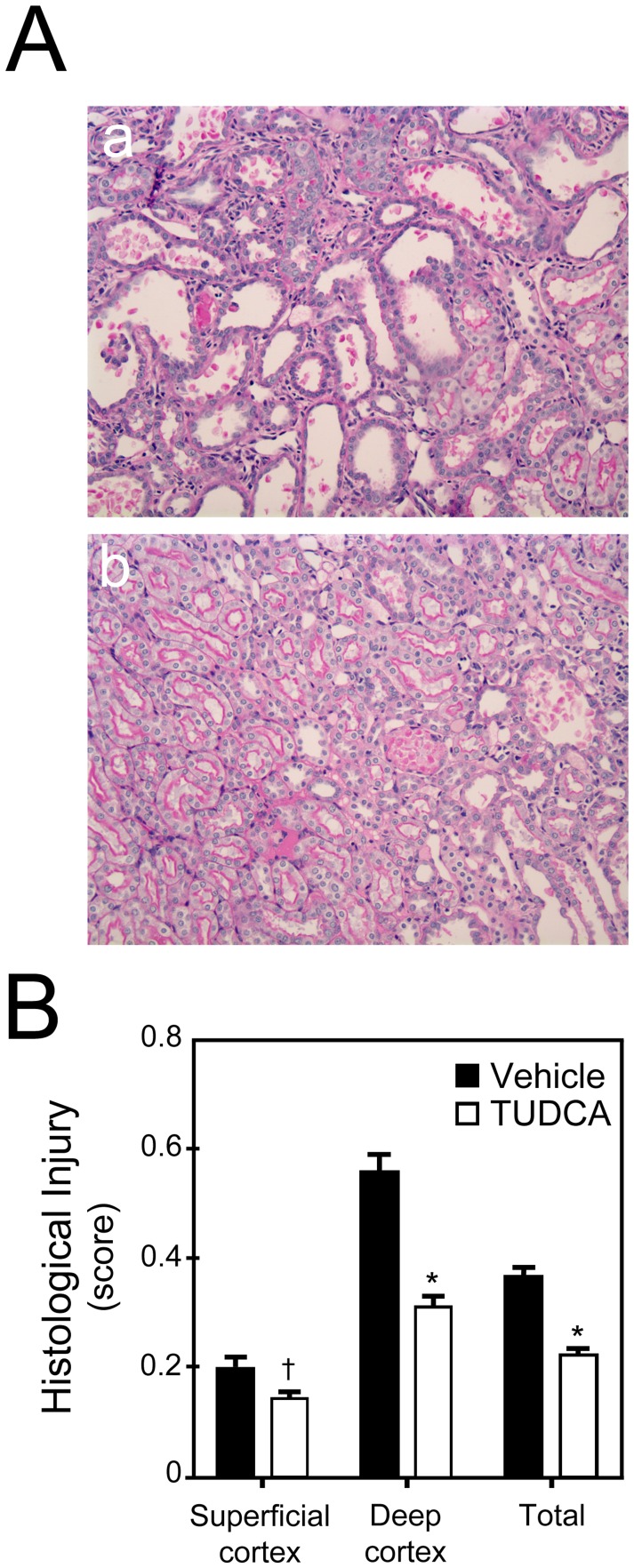
Protection against AKI-induced histological damage by TUDCA. A, Representative PAS stained images from deep cortex from animals that received vehicle control (a) or TUDCA (b). B, Animals that received TUDCA as compared to controls, showed significantly less damage in the deep cortex where the S3 segment is located. Results are expressed as mean ± standard deviation of a least 3 different animals in each group. *p<0.001 and †*p*<0.05 from vehicle-injected controls.

Kidneys were harvested five days following the induction of ischemic AKI. Many proximal tubules from the deep cortex (Figure 2A, a) had significant injury in vehicle-treated rats; in contrast, TUDCA-treated rats had minimal injury (Figure 2A, b). There was significantly less injury in the superficial (*p*<0.05) and deep (*p*<0.001) cortex in TUDCA-treated rats as compared to the vehicle-treated rats (Figure 2B). There were no differences in the medulla or papilla (data not shown).

#### Protection against apoptosis

**Figure 3:**
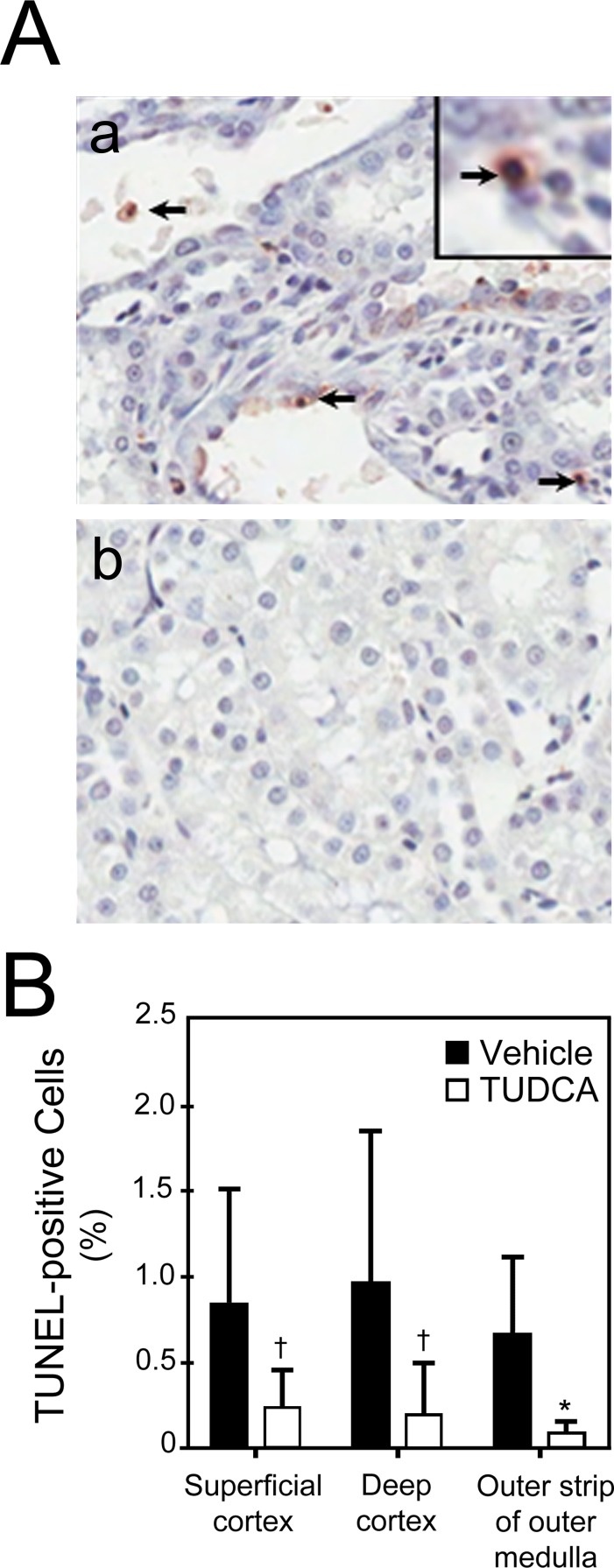
Protection against AKI-induced apoptosis by TUDCA. A, Representative images from cortico-medullary junction from vehicle (a; control) and TUDCA-treated (b) groups. Brown staining and arrows identify TUNEL-positive cells. B, There were significantly less TUNEL-positive cells in the TUDCA-treated group as compared to the vehicle-treated (control) group in the cortex (*p<0.05*) and outer strip of the outer medulla (*p*<0.001). Results are expressed as mean ± standard deviation of a least 3 different animals in each group. *p<0.001 and †*p*<0.05 from vehicle-injected controls.

As TUDCA is a potent anti-apoptotic molecule, we quantified cells undergoing apoptosis in kidney sections by the transferase mediated deoxyuridine triphosphate (dUTP)-digoxigenin nick-end labeling (TUNEL) assay (Figure 3A and B). There were significantly less TUNEL-positive cells in the superficial and deep cortices (*p*<0.05) and outer strip of the outer medulla (*p*<0.001) in the TUDCA-treated rats as compared to the vehicle-treated rats (Figure 3B). The apoptotic cells were exclusively limited to the proximal tubules, which were identified by morphology in the periodic acid Schiff stain (PAS) stained sections.

#### Apoptosis pathway analysis

**Figure 4:**
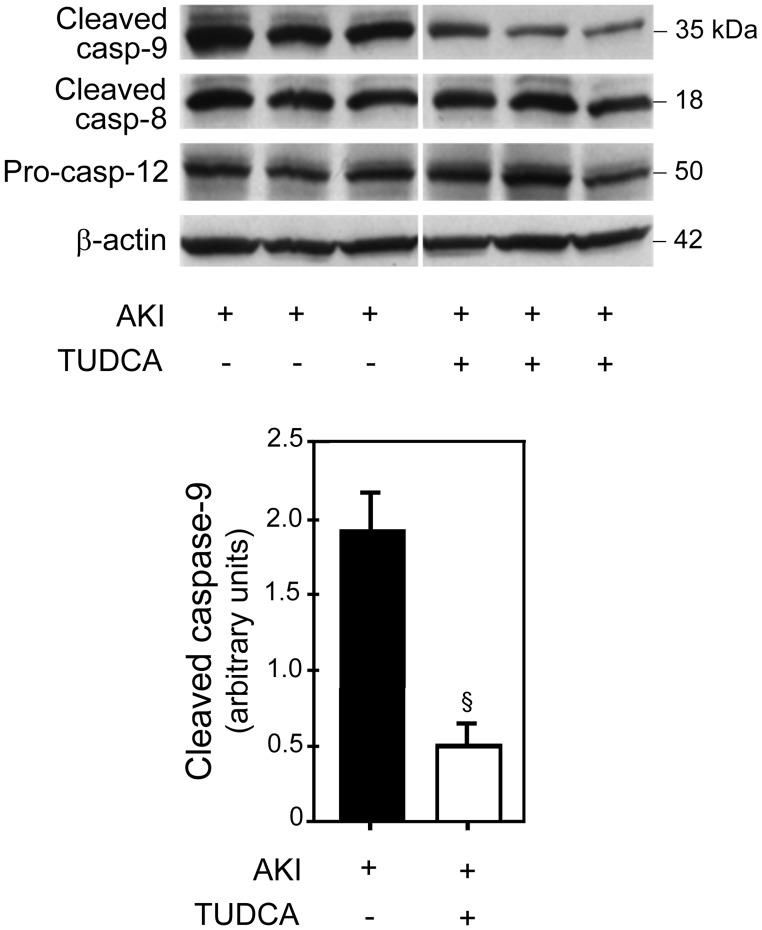
Apoptosis pathway analysis. TUDCA treatment significantly blocked activation of caspase-9 following AKI as compared to vehicle treatment (rats 1, 2, and 3) (top panel). There was no difference in the activation of caspase-8 and caspase-12 between the TUDCA- and vehicle-treated rats. Densitometry analysis of cleaved caspase-9 normalized for β-actin (lower panel). When densitometry results for caspase-9 were compared between the TUDCA- and vehicle-treated groups, there was significantly less (*p*<0.01) activation of caspase-9 in the TUDCA group. Results are expressed as mean ± standard deviation of a least 3 different animals in each group. §*p*<0.01 from vehicle-injected controls.

We determined activation of the mitochondrial, death-receptor, and endoplasmic reticulum (ER)-stress pathways of apoptosis by Western blot analysis for active caspase-9, caspase-8, and caspase-12, respectively. Activation of caspase-9 was significantly inhibited by TUDCA (*p*<0.01) (Figure 4); and the results were confirmed by densitometry using β-actin as the loading control. Interestingly, there was no significant difference in the activation of caspase-8 and caspase-12 between the TUDCA- and vehicle-treated rats.

#### Survival pathway analysis

**Figure 5:**
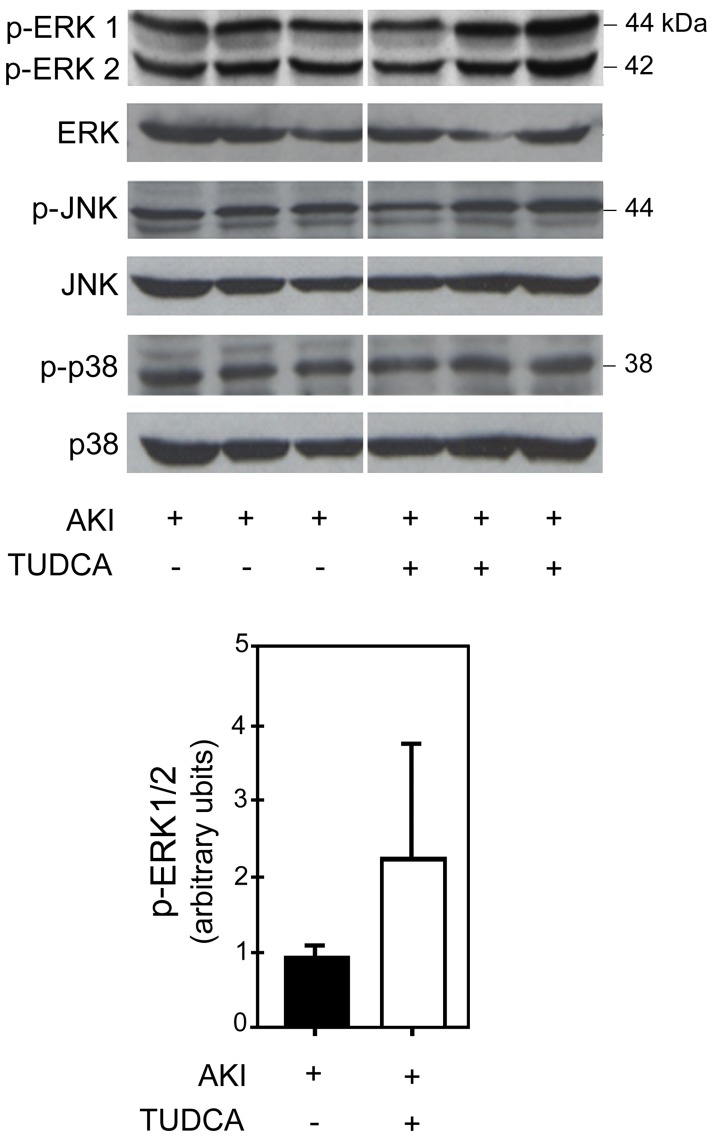
Survival pathway analysis. TUDCA treatment upregulated ERK1/2 following ischemia-reperfusion injury to the kidney in rats (top panel); however, there was no statistical significant difefrence as compared to the vehicle-treated rats (top panel). There was no difference in JNK and p38 proteins between the TUDCA and vehicle groups. Densitometry analysis of ERK1/2 in each rat in the TUDCA group were compared with those in the vehicle group (lower panel); the difference was not significant (*p*  = 0.29). Results are expressed as mean ± standard deviation of a least 3 different animals in each group.

Extracellular regulated kinase 1/2 (ERK1/2), c-Jun N-terminal kinase (JNK), and p38, which are the key components of mitogen-activated protein kinase (MAPK) survival pathway, were analyzed by Western blot. There was increased activation of ERK1/2 in the TUDCA group, although without statistical significance, when compared with controls (Figure 5). No difference was detected in the amount of p-JNK and p-p38 between the two groups.

### Cell Culture Experiments

#### Toxicity studies of TUDCA

**Figure 6:**
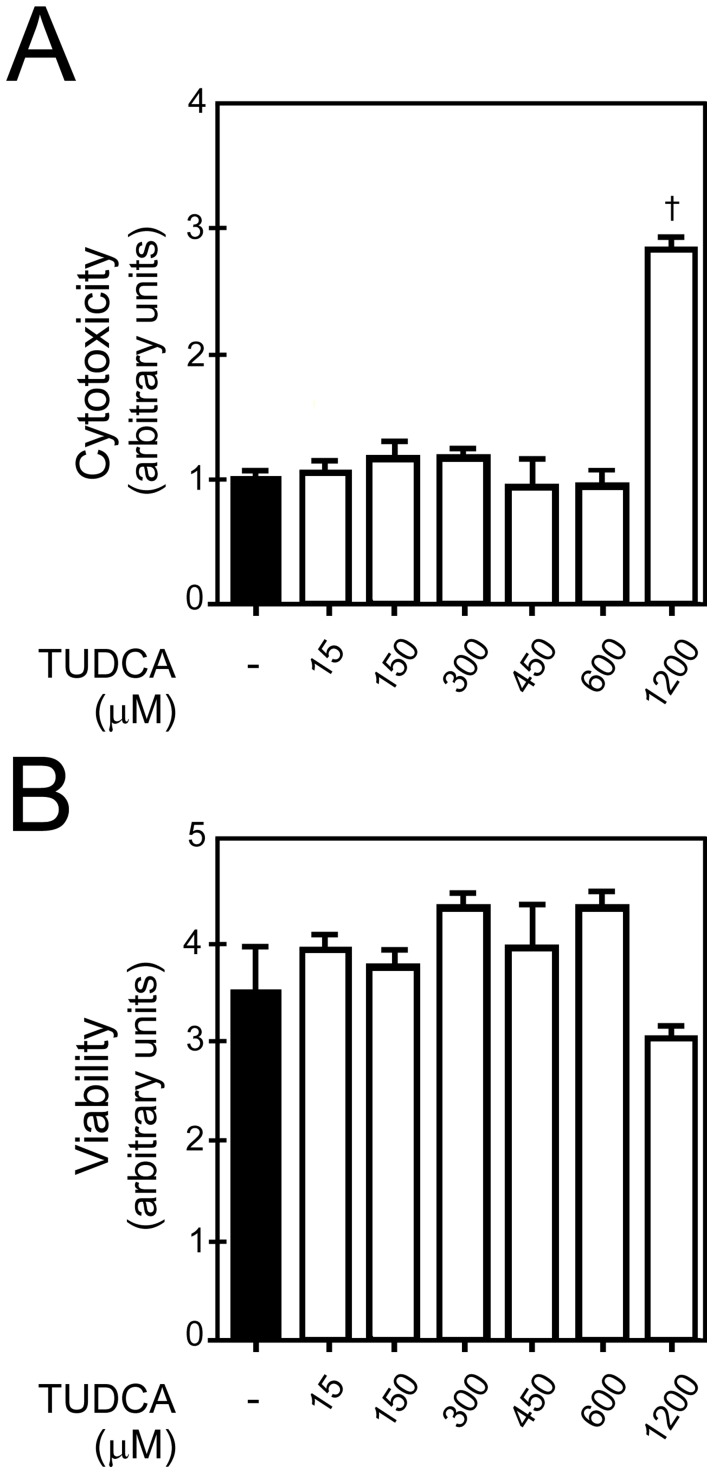
Cytotoxicity studies of TUDCA in primary human RPTE cells. Cells were treated with either vehicle (control) or 15 to 1200 µM of TUDCA for 24 hours. A, TUDCA was not cytotoxic in concentrations from 15 to 600 µM. Significant cytotoxicity was seen only with 1200 µM of TUDCA, as compared to the vehicle (*p*<0.05). B, TUDCA did not decrease cell viability in all the tested concentrations from 15 µM to 1200 µM. Results are expressed as mean ± standard deviation. All experiments were performed in triplicate. †*p*<0.05 from vehicle-treated control.

We treated human renal proximal tubular epithelial cells (RPTE) cells with different concentrations of TUDCA, and performed cytotoxicity and viability assays. TUDCA in concentrations from 15 to 600 µM did not cause cytotoxicity; only 1200 µM of TUDCA was cytotoxic (*p*<0.05) (Figure 6A). None of the tested concentrations of TUDCA decreased cell viability (Figure 6B). We chose concentrations up to 600 µM of TUDCA for subsequent experiments.

#### Protection against apoptosis

**Figure 7:**
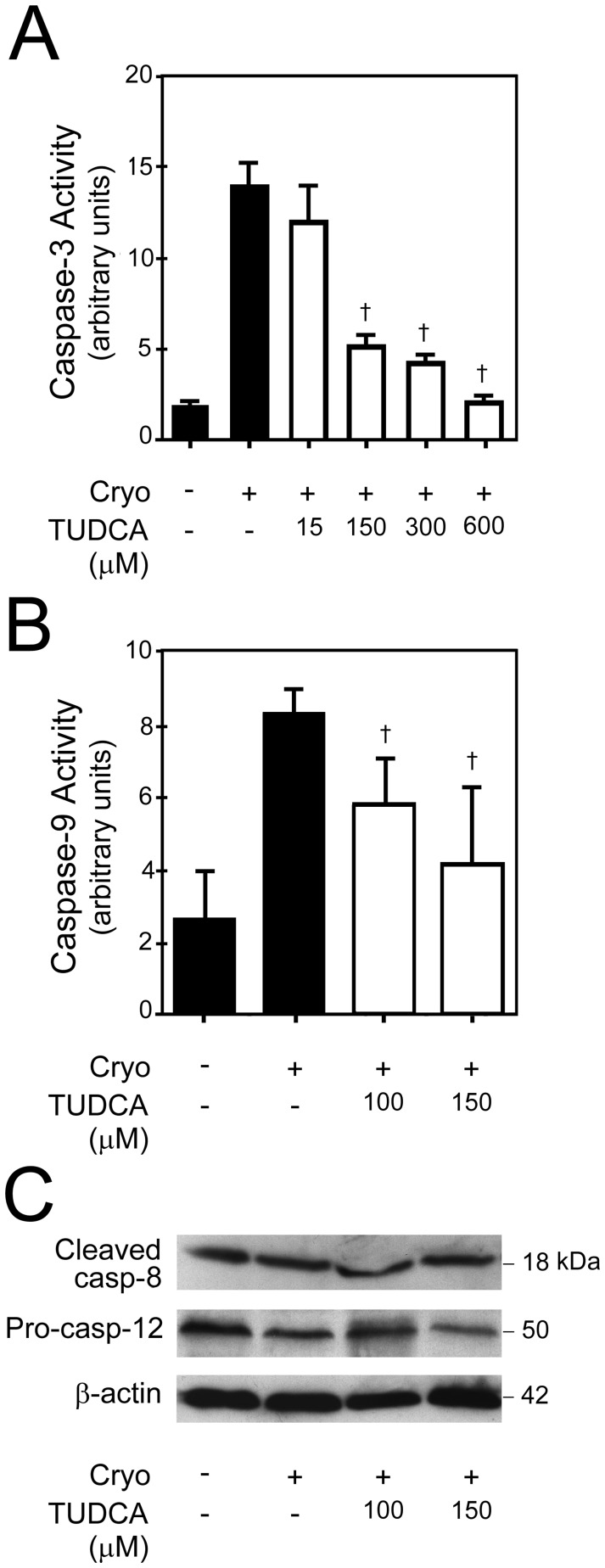
Caspase activation following cryoinjury and treatment with TUDCA in primary human RPTE cells. A, Caspase-3 activity following cryoinjury in RPTE cells treated with either vehicle or different concentrations of TUDCA. Caspase-3 activity in cryoinjured RPTE cells was compared with that in uninjured RPTE cells. There was signficant activation of caspase-3 following cryoinjury (*p*<0.05), which was significantly inhibited by 150–600 µM of TUDCA in a dose-dependent fashion. 15 µM of TUDCA did not inhibit activation of caspase-3 following cryoinjury. B, Caspase-9 activity following cryoinjury in RPTE cells treated with either vehicle or different concentrations of TUDCA. There was statistically significant increased caspase-9 activity in cryoinjured cells as compared to uninjured cells (*p*<0.05). Both 100 and 150 µM of TUDCA significantly decreased caspase-9 activity. C, Caspase-8 and caspase-12 analysis following cryoinjury in RPTE cells treated with either vehicle or different concentrations of TUDCA. There was no difference in the amount of caspase-8 and procaspase-12 between the uninjured and cryoinjured cells treated with vehicle or TUDCA. Results are expressed as mean ± standard deviation. All experiments were performed in triplicate. †*p*<0.05 from vehicle-treated control. Cryo, cryoinjury.

We determined activation of the final common pathway of apoptosis by caspase-3 activity assay. Cryopreservation injury significantly activated caspase-3 in RPTE cells (*p*<0.01), which was significantly inhibited by 150–600 µM of TUDCA in a dose-dependent fashion (*p*<0.05) (Figure 7A). Activation of the mitochondrial pathway of apoptosis was determined via caspase-9 activity assay (Figure 7B). Cryopreservation injury consistently activated caspase-9 in RPTE cells (*p*<0.01), which was significantly inhibited by 100 and 150 µM of TUDCA (*p*<0.05). Next, we determined the activation of death receptor and ER-stress pathways of apoptosis by performing Western blot analysis for caspase-8 and procaspase-12 (Figure 7C). There was no activation of procaspase-8 and procaspase-12 in our model of cryopreservation injury, and TUDCA had no effect on the activation. Thus, TUDCA protected against cryopreservation injury by inhibiting the mitochondrial pathway of apoptosis.

#### Survival pathway analysis

**Figure 8:**
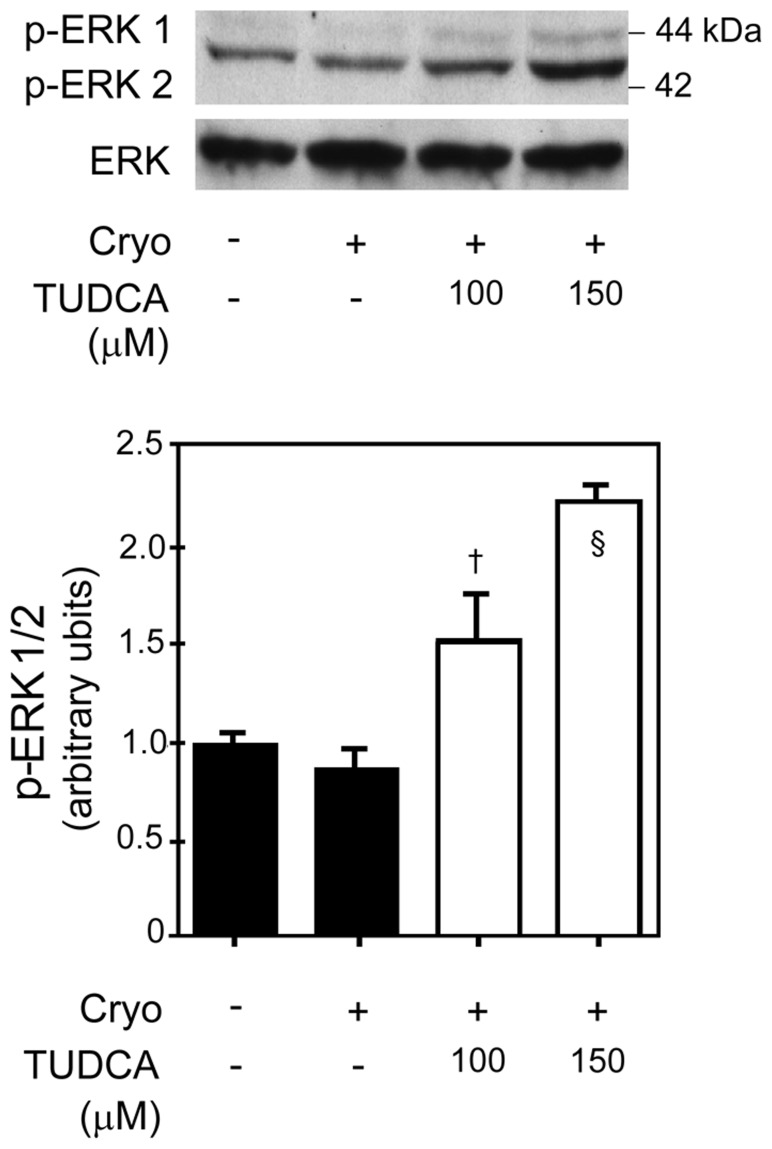
Survival pathway analysis following cryoinjury and treatment with TUDCA in primary human RPTE cells. Phosphorylated ERK1/2 protein in cryoinjured cells that were treated with either vehicle (control) or 100 µM or 150 µM TUDCA as compared to uninjured cells (top panel). There was no difference in the amount of phosphorylated ERK1/2 between uninjured and cryoinjured cells treated with vehicle. Densitometry analysis of phosphorylated ERK1/2 (lower panel). Results are expressed as mean ± standard deviation. All experiments were performed in triplicate. §*p*<0.01 and †*p*<0.05 from vehicle-treated control. Cryo, cryoinjury.

We performed Western blot analysis for active forms of the MAPKs (ERK, JNK, and p38) to determine activation of survival pathways. Treatment with 100 and 150 µM of TUDCA activated ERK1/2 (Figure 8; *p*<0.05 and *p*<0.01, respectively); however, there was no effect of TUDCA on JNK or p38 (data not shown). Thus, upregulation of ERK1/2 by TUDCA contributed to protection against cryopreservation injury.

## Discussion

This is the first study to determine the protective properties of TUDCA against AKI and sets the stage for developing relevant therapies for clinical use. We chose two models of AKI to simulate commonly encountered clinical scenarios: 1) the warm ischemia reperfusion model of AKI in rats recapitulates clinical AKI in the native kidney due to poor perfusion; and 2) the cellular model of cryopreservation injury recapitulates cryopreservation-associated AKI in the donor kidney. We preferred a cellular model to in vivo models of cryopreservation injury to prevent systemic and donor factors such as immunity, inflammation, and donor age from confounding the results.

Warm ischemia reperfusion injury for 45 minutes at 37°C produced a very consistent injury in the rat model. The peak rise in blood urea occurred 24 hours following bilateral renal artery clamping in both the vehicle- and TUDCA-treated rats. As compared to rats treated with vehicle, rats treated with TUDCA had significantly less peak elevation in blood urea following AKI. Blood urea values subsequently declined until day 4, when they plateaued in the vehicle group, while continued to improve in the TUDCA group until the day of euthanasia. Thus, TUDCA reduced the severity of AKI without postponing its onset.

The functional protection against AKI by TUDCA was supported by less severe histological injury seen in kidneys of TUDCA-treated rats. The deep cortex, where the S3 segment of the proximal tubule is located and which sustains maximum injury following ischemia-reperfusion, was better preserved in the TUDCA group. Furthermore, like several published studies in other organs, TUDCA provided protection against apoptosis following AKI. There were significantly less TUNEL-positive cells in the superficial and deep cortices and in the outer strip of the outer medulla in the TUDCA-treated rats as compared to the vehicle-treated rats. Based on the morphological appearance, the TUNEL-positive cells were present exclusively in the proximal tubules. We did not find apoptotic cells in the distal tubules following AKI as reported by other investigators [32], [33]. Thus, these results were consistent with the published studies on the protection by TUDCA against acute injury in animal models of stroke and spinal cord injury [18], [29]. It would also be interesting to test the protective effect of TUDCA in non-hemodynamic injury-mediated AKI.

Activation of caspase-9, which represents the mitochondrial pathway of apoptosis, was significantly inhibited by TUDCA following AKI. TUDCA has been shown to inhibit the mitochondrial pathway of apoptosis in primary hepatocytes, neurons, and in animal models of ischemic injury such as stroke [15], [17], [18], [34]. Similar to the mitochondrial pathway of apoptosis, the ER-stress pathway of apoptosis plays an important role in the pathogenesis of glomerular, tubular, and interstitial kidney diseases [35]. In particular, following ischemic kidney injury, investigators have shown activation of the ER-stress pathway of apoptosis and protection by its inhibitors [36], [37]. In contrast to the mitochondrial pathway, in our model, TUDCA did not have any effect on the ER-stress and death receptor pathways of apoptosis. This is somewhat surprising in light of recent studies demonstrating the ability of TUDCA to reduce ER-stress induced caspase-12 activation [13], [38]–[41].

MAPKs constitute important survival pathways in response to several stimuli in mammals, which include JNK, p38, and ERK. Activation of JNK and p38 has been shown to facilitate cell death, while that of ERK1/2 to promote cell survival following acute injury [42]. Furthermore, ischemic preconditioning of kidneys, which is protective against AKI, acts through activation of ERK1/2 [43]. TUDCA has been shown to inhibit apoptosis in hepatocytes and following acute stroke in rats by activating survival pathways [15], [17]. In our model of AKI, TUDCA increased ERK1/2 levels in two out of three rats. TUDCA had no effect on JNK and p38 pathways. Although this finding is encouraging, it needs further confirmation before one can conclude that activation of ERK1/2 by TUDCA following AKI plays a protective role.

Similar to native kidneys, AKI in the donor kidney is a significant clinical problem. Cessation of blood supply following harvesting results in AKI. To minimize this risk, donor kidneys are currently cryopreserved in specialized solutions such as University of Wisconsin solution. Although a major advancement in the field, the current cryopreservation techniques still results in significant graft injury [44]. Cryopreservation injury to the donor kidney leads to increased incidence of delayed graft function, acute and chronic rejection, and poor short- and long-term graft outcome [45]–[49]. Furthermore, the current cryopreservation time in the USA has remained long at approximately 21 hours. Therefore, there is a pressing need to improve the current cryopreservation techniques. The current studies were performed to set the stage for developing improved cryopreservation solutions for clinical use.

Kidney cells die by necrosis during cryopreservation and by apoptosis during warm reperfusion following transplantation [50]. There is activation of apoptosis pathways during cryopreservation [51], and survival pathways play an important role in resisting cell death [52]–[54]. Furthermore, TUDCA has been shown to reduce structural injury to human livers following cryopreservation; however, in this study, the mechanism of protection was not studied [22].

In the current study, we investigated the efficacy and mechanisms of action of TUDCA against cryopreservation injury. TUDCA was not cytotoxic to RPTE cells in concentrations up to 600 µM and it did not decrease cell viability in all the tested concentrations. Although TUDCA has been shown to be safe in concentrations ranging from 100 nM up to 5 mM in cell culture experiments, most studies have studied 100 to 500 µM of TUDCA [34], [38]–[40], [55]. Our chosen concentrations of 100 and 150 µM of TUDCA were similar to those used by other investigators.

Caspase-3 is activated following cryopreservation of cells and caspase inhibitor provides protection [56]. Similar to the published literature, caspase-3 was consistently activated following cryopreservation injury to the RPTE cells, and was inhibited by TUDCA in a dose-dependent fashion. Similarly, there was activation of mitochondrial pathway of apoptosis in our model of cryopreservation injury, and 100 and 150 µM of TUDCA provided protection [57]–[60]. This is an advancement of the previously known anti-apoptotic properties of TUDCA in models of warm ischemia-reperfusion injury [16], [61]. Unlike the mitochondrial pathway of apoptosis, there was no activation of the ER-stress and death receptor pathway of apoptosis following cryopreservation injury. Further, ERK1/2 survival pathways were activated by both 100 and 150 µM of TUDCA following cryopreservation injury to the RPTE cells, similarly to upregulation of ERK1/2 seen in two of three rats in the in vivo model of AKI. This is the first report of activation of ERK1/2 pathways by TUDCA following cryopreservation injury. Much like in the *in vivo* model, there was no effect of TUDCA on JNK and p38 pathways following cryopreservation injury.

In conclusion, TUDCA was protective in the rat model of ischemia-reperfusion induced AKI and cellular model of cryopreservation injury. It provided protection in the tested models of AKI by inhibiting the mitochondrial pathway of apoptosis and upregulating ERK1/2 survival pathways. Results of this study and a proven safety profile of TUDCA in humans will open the door for conducting human feasibility studies in patients with AKI, an important area of investigation that currently lacks effective therapy. We anticipate administration of TUDCA prior to precipitating events will prevent AKI in humans.

## Materials and Methods

### Ethics Statement

All animal work was approved by the Institutional Animal Care and Use Committee at the University of Minnesota (Minneapolis, MN) and received humane care according to the criteria outlined in the “Guide for the Care and Use of Laboratory Animals” prepared by the National Academy of Sciences and published by the National Institutes of Health (NIH publication 86-23; revised 1985).

### Materials

Normal human RPTE cells were purchased from Lonza Inc. (Walkersville, MD). The University of Wisconsin solution was obtained from BTL solutions LLC (Columbia, SC). TUDCA and Cell Lytic MT Mammalian Tissue Lysis/Extraction Reagent were purchased from Sigma (St Louis, MO). Protease Inhibitor Cocktail and Bicinchoninic Acid Protein Assay kits were purchased from Pierce Biotechnology Inc (Rockford, IL). 12% Tris-HCl sodium dodecyl sulfate-polyacrylamide gel electrophoresis (SDS-PAGE) pre-cast gel was obtained from Bio-Rad Laboratories Inc (Hercules, CA). Amersham Hybond ECL nitrocellulose membrane, Amersham hyperfilm and peroxidase-labeled anti-mouse/rabbit IgG were purchased from GE Healthcare (Waukesha, WI). Anti-phospho-ERK1/2 antibody was purchased from New England BioLabs (Boston, MA); anti-phospho-p38 and anti-phospho-JNK from Santa Cruz Biotechnology Inc. (Santa Cruz, CA); anti-caspase-8 and anti-caspase-12 from BioVision Inc. (Mountainview, CA); anti-caspase-9 antibody from Enzo Life Sciences (Plymouth Meeting, PA); and anti-mouse β-actin from Calbiochem (Spring Valley,CA). SuperSignal West Femto Maximum Sensitivity Substrate Kit was from Thermo Fisher Scientific (Waltham, MA). MultiTox-Glo Multiplex Cytotoxicity Assay and Caspase-9 Glo Assay kits were purchased from Promega Corp. (Madison, WI). Apo-One® Homogeneous Caspase-3/7 Assay kit and Caspase-9 Assay kit were purchased from Promega (Madison, WI). Male Sprague Dawley rats were purchased from Harlan Laboratories (Indianapolis, IN). QuantiChromTM Urea Assay kit was purchased from BioAssay Systems (Hayward, CA), APO-DIRECT™ kit was purchased from BD Pharmingen (San Diego,CA). Reflex Clips Applier and Reflex 9 mm Clips were obtained from World Precision Instruments, Inc (Sarasota, FL). 4-0 absorbable sutures were purchased from Ethicon, Johnson and Johnson (Somerville, NJ).

### In vivo Experiments

#### Rat model of AKI

All experiments were performed in accordance with the Institutional Animal Care and Use Committee. Six to eight week old Sprague-Dawley rats were anaesthetized by isoflurane gas, a midline abdominal incision was made, and bilateral renal pedicles were clamped for 45 minutes maintaining body temperature at 37°C. After removing the clamps, abdomen was closed in two layers by using 4-0 absorbable sutures and Reflex 9 mm clips. Blood samples were obtained daily by tail vein puncture. Blood urea levels were measured by the improved Jung method using the QuantiChromTM Urea Assay Kit as per manufacturer’s protocol. Kidneys were harvested five days following surgery.

#### Administration of TUDCA

200 mg/ml of TUDCA (Sigma) was dissolved in phosphate buffered saline at pH 7.5. 400 mg/kg of TUDCA or equal volume of vehicle was administered to rats by daily intraperitoneal injection from three days prior to surgery (day 3) to five days (day 5) following surgery. The TUDCA dose was based on previous studies [17], [18] and its solubility at physiological pH.

#### Histology and TUNEL assay

4% paraformaldehyde-fixed, paraffin-embedded 5 µm kidney sections were stained with PAS stain using standard methods. Histological examination was performed by a renal pathologist in a blinded fashion. Histological injury was scored based on the percentage of tubular cell necrosis, dilation, and cell detachment as per published protocol [62]. In brief, the following criteria were used: 0, no abnormality; 1+, changes affecting less than 25% of sample; 2+, changes affecting 25%–50%; 3+, changes affecting 50%–75%; and 4+, changes affecting >75% of the sample. Apoptotic cells were detected by TUNEL assay using APO-DIRECT™ kit as per manufacturer’s protocol. The average injury score and percentage of TUNEL-positive cells from the renal cortex (superficial and deep), medulla (outer and inner), and papilla were counted and calculated separately and averaged to obtain the total score. At least 10 fields (under X 200 magnification) were reviewed at each location.

### Cell Culture Experiments

#### Cell culture

RPTE cells were grown in Renal Epithelial Cell Basal Medium (REBM) with full supplements at 37°C in 5% CO_2_ incubator as per supplier’s instructions. RPTE cells were able to proliferate for 6–8 passages under the culture conditions.

#### Cryopreservation injury

We have utilized a published cell culture model of cryopreservation injury [51], [63]. In brief, RPTE cells were grown to 80% confluence in the complete medium containing TUDCA or vehicle. The complete medium was then replaced with UW solution containing TUDCA or vehicle. The culture plates were subsequently incubated in a temperature-regulated refrigerator at 4°C for 48 hours. To simulate warm reperfusion phase of kidney transplantation, UW solution was replaced with complete medium containing TUDCA or vehicle, and cells were cultured for an additional 24 hours at 37°C. We used 100 or 150 µM TUDCA for these experiments.

#### Viability and cytotoxicity assays

Cytotoxicity and viability were determined using MultiTox-Glo Multiplex Cytotoxicity Assay kit as per manufacturer’s protocols. RPTE cells were seeded in 96-well culture plates at a density of 1.2×10^4^ cells per well. Subsequently, TUDCA was added to the wells to achieve final concentrations of 15, 150, 300, 450, 600, and 1200 µM. Following 24 hours of culture, to determine viability, 50 µL of GF-AFC Reagent was added to each well. The plates were gently shaken and incubated at 37°C for 30 minutes in the dark. The cell viability was determined by measuring fluorescence at 400 nmEx/505 nmEm. Subsequently, to determine cytotoxicity, 50 µL of AAF-Glo Reagent was added to each well. The plates were shaken gently and incubated at room temperature for 15 minutes in the dark. Cytotoxicity was determined by measuring luminescence as per manufacturer’s protocol.

### Other Methods

#### Protein extraction

Frozen kidney tissue was ground in liquid nitrogen using a pestle and mortar. One ml of Tissue Protein Extraction Reagent with 1× protease and phosphatase inhibitor was added to the ground kidney tissue (per 30 mg of tissue) or RPTE cells (per 1.2×10^6^ cells). The lysate was incubated at 4°C for 10 minutes with vigorous shaking and subsequently centrifuged at 4°C for 10 minutes at 13,000 g. The resultant supernatant was immediately frozen in liquid nitrogen and stored at −80°C until further analysis. The amount of protein present in the solution was quantified by the Bicinchoninic Acid Protein Assay kit as per manufacturer’s protocol.

#### Western blot analysis

20 µg of protein was mixed with SDS-PAGE sample buffer, boiled for 5 minutes, electrophoresed on a 12% Tris-HCl SDS-PAGE gel for 1 hour at 200 V, and electroblotted onto nitrocellulose membrane. The membrane was blocked for 1 hour at room temperature in 1× Tris-buffered saline with 0.1% Tween-20 at pH 7.4 containing 5% dry milk powder. To detect p-ERK1/2, p-JNK, p-p38, caspase-8, caspase-9, and caspase-12, the membranes were incubated overnight with respective primary antibodies at 4°C and appropriate secondary antibodies at room temperature for one hour. The immunoblot was detected using SuperSignal West Femto Maximum Sensitivity Substrate Kit. To verify equal loading of proteins, the membrane was stripped at room temperature with 1× Tris-buffered saline at pH 2.5 for 30 minutes and re-probed with the mouse anti-β-actin antibody and corresponding secondary antibody.

#### Caspase-3 activity assay

The caspase-3 activity in kidney and RPTE extracts was quantified using Apo-One® Homogeneous Caspase-3/7 Assay kit as per the manufacturer’s protocol. In brief, 100 µg of protein in 100 µL of lysis buffer was added to 100 µL of the assay buffer containing non-fluorescent caspase-3 substrate, bis-N-CBZL-aspartyl-L-glutamyl-L-valyl-L-aspartic acid amide (Z-DEVD-R110). The mixture was then incubated for 1 hour at 30°C during which Z-DEVD-R110 was converted into a fluorescent substrate by the active caspase-3 enzyme. Fluorescence was measured by Spectramax M12 fluorescent plate reader (Molecular Devices) using wavelengths of 485 nmEx/520 nmEm. The fluorescent signal was expressed in relative fluorescent units.

#### Caspase-9 assay

The caspase-9 activity was measured in RPTE cells using Caspase-9 Assay kit. RPTE cells were suspended in 50 µL of chilled Cell Lysis Buffer and incubated on ice for 10 minutes. Subsequently, 50 µL of 2× Reaction Buffer and 5 µL of 1 mM LEHD-AFC substrate was added to each samples and the mixture was incubated at 37°C for 2 hours. The caspase-9 activity was quantified by measuring luminescence.

### Statistical Analysis

Data were expressed as mean and standard deviation unless otherwise stated. The differences between normally distributed data were analyzed by independent Student’s T-test. Nonparametric unpaired Mann-Whitney test was used if the data was not normally distributed. Multiple group comparisons were performed using ANOVA with post-test according to Bonferroni. A *p* value of less than 0.05 was considered statistically significant.

## References

[1] Soni SS, Ronco C, Katz N, Cruz DN (2009) Early diagnosis of acute kidney injury: the promise of novel biomarkers. Blood Purif 28: 165–174. 19590184 10.1159/000227785

[2] Ishani A, Xue JL, Himmelfarb J, Eggers PW, Kimmel PL, et al (2009) Acute kidney injury increases risk of ESRD among elderly. J Am Soc Nephrol 20: 223–228. 19020007 10.1681/ASN.2007080837 PMC2615732

[3] Finkenstaedt JT, Merrill JP (1956) Renal function after recovery from acute renal failure. N Engl J Med 254: 1023–1026. 13322205 10.1056/NEJM195605312542203

[4] Lowe KG (1952) The late prognosis in acute tubular necrosis; an interim follow-up report on 14 patients. Lancet 1: 1086–1088. 14928581 10.1016/s0140-6736(52)90744-7

[5] Venkatachalam MA, Griffin KA, Lan R, Geng H, Saikumar P, et al (2010) Acute kidney injury: a springboard for progression in chronic kidney disease. Am J Physiol Renal Physiol 298: F1078–1094. 20200097 10.1152/ajprenal.00017.2010 PMC2867413

[6] Kindt N, Menzebach A, Van de Wouwer M, Betz I, De Vriese A, et al (2008) Protective role of the inhibitor of apoptosis protein, survivin, in toxin-induced acute renal failure. FASEB J 22: 510–521. 17804696 10.1096/fj.07-8882com

[7] Hausenloy DJ, Yellon DM (2006) Survival kinases in ischemic preconditioning and postconditioning. Cardiovasc Res 70: 240–253. 16545352 10.1016/j.cardiores.2006.01.017

[8] Park KM (2001) Prevention of kidney ischemia/reperfusion-induced functional injury and jnk, p38, and MAPK kinase activation by remote ischemic pretreatment. J Biol Chem 276: 11870–11876. 11150293 10.1074/jbc.M007518200

[9] Chien CT, Shyue SK, Lai MK (2007) Bcl-xL augmentation potentially reduces ischemia/reperfusion induced proximal and distal tubular apoptosis and autophagy. Transplantation 84: 1183–1190. 17998875 10.1097/01.tp.0000287334.38933.e3

[10] Ortiz A, Justo P, Catalan MP, Sanz AB, Lorz C, et al (2002) Apoptotic cell death in renal injury: The rationale for intervention. Curr Drug Targets Immune Endocr Metabol Disord 2: 181–192. 12476791

[11] Rodrigues CM, Ma X, Linehan-Stieers C, Fan G, Kren BT, et al (1999) Ursodeoxycholic acid prevents cytochrome c release in apoptosis by inhibiting mitochondrial membrane depolarization and channel formation. Cell Death Differ 6: 842–854. 10510466 10.1038/sj.cdd.4400560

[12] Rodrigues CM, Fan G, Ma X, Kren BT, Steer CJ (1998) A novel role for ursodeoxycholic acid in inhibiting apoptosis by modulating mitochondrial membrane perturbation. J Clin Invest 101: 2790–2799. 9637713 10.1172/JCI1325 PMC508870

[13] Xie Q, Khaoustov VI, Chung CC, Sohn J, Krishnan B, et al (2002) Effect of tauroursodeoxycholic acid on endoplasmic reticulum stress-induced caspase-12 activation. Hepatology 36: 592–601. 12198651 10.1053/jhep.2002.35441

[14] Azzaroli F, Mehal W, Soroka CJ, Wang L, Lee J, et al (2002) Ursodeoxycholic acid diminishes fas-ligand-induced apoptosis in mouse hepatocytes. Hepatology 36: 49–54. 12085348 10.1053/jhep.2002.34511

[15] Schoemaker MH, Conde de la Rosa L, Buist-Homan M, Vrenken TE, Havinga R, et al (2004) Tauroursodeoxycholic acid protects rat hepatocytes from bile acid-induced apoptosis via activation of survival pathways. Hepatology 39: 1563–1573. 15185297 10.1002/hep.20246

[16] Rivard AL, Steer CJ, Kren BT, Rodrigues CM, Castro RE, et al (2007) Administration of tauroursodeoxycholic acid (TUDCA) reduces apoptosis following myocardial infarction in rat. Am J Chin Med 35: 279–295. 17436368 10.1142/S0192415X07004813

[17] Rodrigues CM, Sola S, Nan Z, Castro RE, Ribeiro PS, et al (2003) Tauroursodeoxycholic acid reduces apoptosis and protects against neurological injury after acute hemorrhagic stroke in rats. Proc Natl Acad Sci U S A 100: 6087–6092. 12721362 10.1073/pnas.1031632100 PMC156330

[18] Rodrigues CM, Spellman SR, Sola S, Grande AW, Linehan-Stieers C, et al (2002) Neuroprotection by a bile acid in an acute stroke model in the rat. J Cereb Blood Flow Metab 22: 463–471. 11919517 10.1097/00004647-200204000-00010

[19] Hagey LR, Crombie DL, Espinosa E, Carey MC, Igimi H, et al (1993) Ursodeoxycholic acid in the Ursidae: Biliary bile acids of bears, pandas, and related carnivores. J Lipid Res 34: 1911–1917. 8263415

[20] Sola S, Garshelis DL, Amaral JD, Noyce KV, Coy PL, et al (2006) Plasma levels of ursodeoxycholic acid in black bears, Ursus Americanus: Seasonal changes. Comp Biochem Physiol C Toxicol Pharmacol 143: 204–208. 16571381 10.1016/j.cbpc.2006.02.002

[21] Arat M, Idilman R, Soydan EA, Soykan I, Erden E, et al (2005) Ursodeoxycholic acid treatment in isolated chronic graft-vs-host disease of the liver. Clin Transplant 19: 798–803. 16313328 10.1111/j.1399-0012.2005.00424.x

[22] Falasca L, Tisone G, Palmieri G, Anselmo A, Di Paolo D, et al (2001) Protective role of tauroursodeoxycholate during harvesting and cold storage of human liver: A pilot study in transplant recipients. Transplantation 71: 1268–1276. 11397961 10.1097/00007890-200105150-00015

[23] Festi D, Montagnani M, Azzaroli F, Lodato F, Mazzella G, et al (2007) Clinical efficacy and effectiveness of ursodeoxycholic acid in cholestatic liver diseases. Curr Clin Pharmacol 2: 155–177. 18690863 10.2174/157488407780598171

[24] Kuiper EM, Hansen BE, de Vries RA, den Ouden-Muller JW, van Ditzhuijsen TJ, et al (2009) Improved prognosis of patients with primary biliary cirrhosis that have a biochemical response to ursodeoxycholic acid. Gastroenterology 136: 1281–1287. 19208346 10.1053/j.gastro.2009.01.003

[25] Omata M, Yoshida H, Toyota J, Tomita E, Nishiguchi S, et al (2007) A large-scale, multicentre, double-blind trial of ursodeoxycholic acid in patients with chronic hepatitis C. Gut. 56: 1747–1753. 10.1136/gut.2007.120956 PMC2095694 17573387

[26] Paumgartner G, Beuers U (2002) Ursodeoxycholic acid in cholestatic liver disease: Mechanisms of action and therapeutic use revisited. Hepatology 36: 525–531. 12198643 10.1053/jhep.2002.36088

[27] Sola S, Aranha MM, Steer CJ, Rodrigues CM (2007) Game and players: Mitochondrial apoptosis and the therapeutic potential of ursodeoxycholic acid. Curr Issues Mol Biol 9: 123–138. 17489439

[28] Amaral JD, Viana RJS, Ramalho RM, Steer CJ, Rodrigues CMP (2009) Bile acids: Regulation of apoptosis by ursodeoxycholic acid. J Lipid Res 50: 1721–1734. 19417220 10.1194/jlr.R900011-JLR200 PMC2724780

[29] Colak A, Kelten B, Sagmanligil A, Akdemir O, Karaoglan A, et al (2008) Tauroursodeoxycholic acid and secondary damage after spinal cord injury in rats. J Clin Neurosci 15: 665–671. 18343118 10.1016/j.jocn.2007.06.002

[30] Duan WM, Rodrigues CM, Zhao LR, Steer CJ, Low WC (2002) Tauroursodeoxycholic acid improves the survival and function of nigral transplants in a rat model of parkinson's disease. Cell Transplant 11: 195–205. 12075985

[31] Kars M, Yang L, Gregor MF, Mohammed BS, Pietka TA, et al (2010) Tauroursodeoxycholic acid may improve liver and muscle but not adipose tissue insulin sensitivity in obese men and women. Diabetes 59: 1899–1905. 20522594 10.2337/db10-0308 PMC2911053

[32] Hall AM, Unwin RJ, Parker N, Duchen MR (2009) Multiphoton imaging reveals differences in mitochondrial function between nephron segments. J Am Soc Nephrol 20: 1293–1302. 19470684 10.1681/ASN.2008070759 PMC2689904

[33] Beeri R, Symon Z, Brezis M, Ben-Sasson SA, Baehr PH, et al (1995) Rapid DNA fragmentation from hypoxia along the thick ascending limb of rat kidneys. Kidney Int 47: 1806–1810. 7543962 10.1038/ki.1995.249

[34] Rodrigues CM, Stieers CL, Keene CD, Ma X, Kren BT, et al (2000) Tauroursodeoxycholic acid partially prevents apoptosis induced by 3-nitropropionic acid: Evidence for a mitochondrial pathway independent of the permeability transition. J Neurochem 75: 2368–2379. 11080188 10.1046/j.1471-4159.2000.0752368.x

[35] Inagi R (2009) Endoplasmic reticulum stress in the kidney as a novel mediator of kidney injury. Nephron Exp Nephrol 112: e1–9. 19342868 10.1159/000210573

[36] Bailly-Maitre B, Fondevila C, Kaldas F, Droin N, Luciano F, et al (2006) Cytoprotective gene bi-1 is required for intrinsic protection from endoplasmic reticulum stress and ischemia-reperfusion injury. Proc Natl Acad Sci U S A 103: 2809–2814. 16478805 10.1073/pnas.0506854103 PMC1413773

[37] Bando Y, Tsukamoto Y, Katayama T, Ozawa K, Kitao Y, et al (2004) Orp150/hsp12a protects renal tubular epithelium from ischemia-induced cell death. FASEB J 18: 1401–1403. 15240565 10.1096/fj.03-1161fje

[38] Ozcan U, Yilmaz E, Ozcan L, Furuhashi M, Vaillancourt E, et al (2006) Chemical chaperones reduce ER stress and restore glucose homeostasis in a mouse model of type 2 diabetes. Science 313: 1137–1140. 16931765 10.1126/science.1128294 PMC4741373

[39] Lee YY, Hong SH, Lee YJ, Chung SS, Jung HS, et al (2010) Tauroursodeoxycholate (TUDCA), chemical chaperone, enhances function of islets by reducing ER stress. Biochem Biophys Res Commun 397: 735–739. 20541525 10.1016/j.bbrc.2010.06.022

[40] Chen Y, Liu CP, Xu KF, Mao XD, Lu YB, et al (2008) Effect of taurine-conjugated ursodeoxycholic acid on endoplasmic reticulum stress and apoptosis induced by advanced glycation end products in cultured mouse podocytes. Am J Nephrol 28: 1014–1022. 18648192 10.1159/000148209

[41] Malo A, Krüger B, Seyhun E, Schäfer C, Hoffmann RT, et al (2010) Tauroursodeoxycholic acid reduces endoplasmic reticulum stress, trypsin activation, and acinar cell apoptosis while increasing secretion in rat pancreatic acini. Am J Physiol Gastrointest Liver Physiol 299: G877–886. 20671193 10.1152/ajpgi.00423.2009

[42] Xia Z, Dickens M, Raingeaud J, Davis RJ, Greenberg ME (1995) Opposing effects of ERK and JNK-p38 MAP kinases on apoptosis. Science 270: 1326–1331. 7481820 10.1126/science.270.5240.1326

[43] Park KM, Chen A, Bonventre JV (2001) Prevention of kidney ischemia/reperfusion-induced functional injury and JNK, p38, and MAPK kinase activation by remote ischemic pretreatment. J Biol Chem 276: 11870–11876. 11150293 10.1074/jbc.M007518200

[44] Quiroga I, McShane P, Koo DD, Gray D, Friend PJ, et al. 10.1093/ndt/gfl042 16490743

[45] Bronzatto EJ, da Silva Quadros KR, Santos RL, Alves-Filho G, Mazzali M (2009) Delayed graft function in renal transplant recipients: Risk factors and impact on 1-year graft function: A single center analysis. Transplant Proc 41: 849–851. 19376369 10.1016/j.transproceed.2009.02.004

[46] Jushinskis J, Trushkov S, Bicans J, Suhorukov V, Shevelev V, et al (2009) Risk factors for the development of delayed graft function in deceased donor renal transplants. Transplant Proc 41: 746–748. 19328971 10.1016/j.transproceed.2009.01.037

[47] Mikhalski D, Wissing KM, Ghisdal L, Broeders N, Touly M, et al (2008) Cold ischemia is a major determinant of acute rejection and renal graft survival in the modern era of immunosuppression. Transplantation 85: S3–9. 18401260 10.1097/TP.0b013e318169c29e

[48] Ojo AO, Wolfe RA, Held PJ, Port FK, Schmouder RL (1997) Delayed graft function: Risk factors and implications for renal allograft survival. Transplantation 63: 968–974. 9112349 10.1097/00007890-199704150-00011

[49] Salahudeen AK, Haider N, May W (2004) Cold ischemia and the reduced long-term survival of cadaveric renal allografts. Kidney Int 65: 713–718. 14717946 10.1111/j.1523-1755.2004.00416.x

[50] Burns AT, Davies DR, McLaren AJ, Cerundolo L, Morris PJ, et al (1998) Apoptosis in ischemia/reperfusion injury of human renal allografts. Transplantation 66: 872–876. 9798696 10.1097/00007890-199810150-00010

[51] Salahudeen AK, Huang H, Joshi M, Moore NA, Jenkins JK (2003) Involvement of the mitochondrial pathway in cold storage and rewarming-associated apoptosis of human renal proximal tubular cells. Am J Transplant 3: 273–280. 12614281 10.1034/j.1600-6143.2003.00042.x

[52] Omori K, Valiente L, Orr C, Rawson J, Ferreri K, et al (2007) Improvement of human islet cryopreservation by a p38 MAPK inhibitor. Am J Transplant 7: 1224–1232. 17331110 10.1111/j.1600-6143.2007.01741.x

[53] Omori K, Valiente L, Orr C, Rawson J, Ferreri K, et al (2005) Inhibition of p38 mitogen-activated protein kinase protects human islets from cryoinjury and improves the yield, viability, and quality of frozen-thawed islets. Transplant Proc 37: 3422–3423. 16298615 10.1016/j.transproceed.2005.09.089

[54] Yoshinari D, Takeyoshi I, Kobayashi M, Koyama T, Iijima K, et al (2001) Effects of a p38 mitogen-activated protein kinase inhibitor as an additive to University of Wisconsin solution on reperfusion injury in liver transplantation. Transplantation 72: 22–27. 11468529 10.1097/00007890-200107150-00007

[55] Malo A, Kruger B, Seyhun E, Schafer C, Hoffmann RT, et al (2010) Tauroursodeoxycholic acid reduces endoplasmic reticulum stress, trypsin activation, and acinar cell apoptosis while increasing secretion in rat pancreatic acini. Am J Physiol 299: G877–886. 10.1152/ajpgi.00423.2009 20671193

[56] Stroh C, Cassens U, Samraj AK, Sibrowski W, Schulze-Osthoff K, et al (2002) The role of caspases in cryoinjury: Caspase inhibition strongly improves the recovery of cryopreserved hematopoietic and other cells. FASEB J 16: 1651–1653. 12207004 10.1096/fj.02-0034fje

[57] Anderson CD, Belous A, Pierce J, Nicoud IB, Knox C, et al (2004) Mitochondrial calcium uptake regulates cold preservation-induced Bax translocation and early reperfusion apoptosis. Am J Transplant 4: 352–362. 14961987 10.1111/j.1600-6143.2004.00357.x

[58] Huang J, Nakamura K, Ito Y, Uzuka T, Morikawa M, et al (2005) Bcl-xL gene transfer inhibits Bax translocation and prolongs cardiac cold preservation time in rats. Circulation 112: 76–83. 15983241 10.1161/CIRCULATIONAHA.105.535740

[59] Kuznetsov AV, Schneeberger S, Seiler R, Brandacher G, Mark W, et al (2004) Mitochondrial defects and heterogeneous cytochrome c release after cardiac cold ischemia and reperfusion. Am J Physiol Heart Circ Physiol 286: H1633–1641. 14693685 10.1152/ajpheart.00701.2003

[60] Yagi T, Hardin JA, Valenzuela YM, Miyoshi H, Gores GJ, et al (2001) Caspase inhibition reduces apoptotic death of cryopreserved porcine hepatocytes. Hepatology 33: 1432–1440. 11391532 10.1053/jhep.2001.24560

[61] Ramalho RM, Viana RJ, Castro RE, Steer CJ, Low WC, et al (2008) Apoptosis in transgenic mice expressing the p301l mutated form of human tau. Mol Med 14: 309–317. 18368144 10.2119/2007-00133.Ramalho PMC2274892

[62] Gupta S, Li S, Abedin MJ, Wang L, Schneider E, et al (2010) Effect of notch activation on the regenerative response to acute renal failure. Am J Physiol Renal Physiol 298: F209–215. 19828677 10.1152/ajprenal.00451.2009

[63] Salahudeen AK, Joshi M, Jenkins JK (2001) Apoptosis versus necrosis during cold storage and rewarming of human renal proximal tubular cells. Transplantation 72: 798–804. 11571440 10.1097/00007890-200109150-00010

